# A survey of energy drinks consumption practices among student -athletes in Ghana: lessons for developing health education intervention programmes

**DOI:** 10.1186/1550-2783-9-9

**Published:** 2012-03-24

**Authors:** Christiana Buxton, John E Hagan

**Affiliations:** 1 Department of Science and Mathematics Education (Health Sciences Programme), University of Cape Coast, Cape Coast, Ghana; 2 Department of Health, Physical Education and Recreation, University of Cape Coast, Cape Coast, Ghana

**Keywords:** Energy drinks, Consumption practices, Student-athletes, University

## Abstract

**Background:**

Globally, young adults and college athletes are primary targets of the marketing campaigns of energy drink companies. Consequently, it is reported that young adults and college athletes consume energy drinks frequently. The purpose of this study was to determine the prevalence of energy drink consumption among student-athletes selected from seven public universities in Ghana. The study assessed the energy drink consumption patterns, types usually consumed, frequency of consumption and reasons why athletes consumed energy drinks.

**Methods:**

A total number of 180 student-athletes gave their consent to participate in the study and completed a questionnaire which was administered during an inter-university sports competition.

**Results:**

Most of the participants (62.2%) reported consuming at least one can of energy drink in a week. A high proportion (53.6%) of the respondents who drink energy drinks indicated that they did so to replenish lost energy after training or a competition. Other reasons given as to why energy drinks were consumed by the study participants included to provide energy and fluids to the body (25.9%), to improve performance (9.8%) and to reduce fatigue (5.4%).

**Conclusion:**

These results suggest the need to plan health education programmes to particularly correct some wrong perceptions that athletes have regarding the benefits of energy drinks and also create awareness among student-athletes about the side effects of excessive intake of energy drinks.

## Background

The term "energy drink" refers to soft drinks believed to reduce or prevent fatigue, enhance physical performance, enhance disposition and improve cognitive performance [1]. Energy drinks are frequently consumed by athletes prior to competitions with a view to improving their performance [2]. The belief in energy drinks is held by most athletes, particularly because the term "energy drink" conveys a message that the product has a connection with physical activity. Consequently, an uninformed consumer may assume that some benefits would be derived after consuming these beverages [3]. Paddock [3] indicated that the drive to improve athletic performance and exhibit one's athletic identity could influence student-athletes in particular to consume energy drinks at a relatively higher level than the student population in general. Most energy drinks contain whopping quantities of sugar (up to a quarter of a cup per can) and caffeine, the main active ingredient, although other substances such as taurine, riboflavin, pyridoxine, nicotinamide, B vitamins, and various stimulating herbal derivatives (guarana, ginseng and ginkgo biloba) may be present [4]. The typical high sugar content (usually approximately 9% or 10%) does not only make energy drinks more calorific but also impedes fluid absorption and may lead to abdominal cramping. Caffeine concentrations may range from 70 to 80 milligrams per 8 ounce serving, about three to five times the concentration in cola. However, this has been found to have detrimental health consequences [5]. For instance, Riesenhuber et al. [6] reported that caffeine in energy drinks promotes natriuresis. It also acts as a diuretic agent, resulting in greater fluid losses. Another study revealed that high intakes of caffeine reduces insulin sensitivity [7] and raises the mean arterial blood pressure level of the body [8]. In sum, although caffeine, a component in most energy drinks, provides the consumer with desirable effects such as increased alertness and improved memory, and enhances a person's mood, caffeine also has harmful health consequences as well [1]. For example, energy drinks - such as Red Bull, Lucozade, Rox, Blue Jeans, Gluconade and Burn have become ubiquitous in shops on university campuses. Most athletes consume energy drinks with the hope of obtaining energy, although there is no scientific confirmation of the ergogenic effectiveness of energy drinks [9]. However, one experimental study found out that an intake of energy drinks, compared with a placebo, had energizing effects which were strongest 30 to 60 minutes after consumption, and which were sustained for at least 90 minutes [10].

It has been reported that the consumption of energy drinks, especially among young adults aged between 18 and 25, is currently of great concern [11]. This is because these energy drinks typically contain three times the amount of caffeine present in soft drinks, and in some cases, up to ten times as much. Another issue of great concern is that, for most brands, information regarding the potential negative health effects of an excessive intake is not presented on the labels [12]. Some energy drinks contain ingredients with potential interactions such as between taurine and other amino acids and between caffeine and some herbal extracts. Some herbs combine with caffeine to create a "synergistic effect" which varies from drink to drink [13].

Athletes, particularly those who play highly competitive sports, are more likely to show an interest in new products that assure them of an improvement in their performance or quick recovery after an event. As such they are easily lured to consume these energy beverages. In addition, manufacturers recommend these energy drinks for sports that require high levels of energy such as cross-country and mountain climbing [14]. It has been reported that university and college athletes are usually consumers of energy drinks because they are aggressively marketed to them with messages touting numerous benefits such as an improvement in performance and replenishment of lost energy, among others [3]. For example, it was revealed in a survey of adolescent athletes, that some, as young as 11 years, reported they depended on energy drinks to improve their sports performance [15].

In some developed countries, some reported deaths have been linked to excessive intake of energy drinks. Therefore some governments have instituted restrictions on their importation and sale. For example, countries like France, Turkey, Denmark, Norway, Uruguay and Iceland have banned high-caffeine and taurine energy drinks altogether from the market. Other countries such as Sweden only permit the sale of energy drinks in pharmaceutical shops as medicinal products. In other countries, such as Canada, it is required that warning labels clearly caution against their use by children or pregnant women, consumption in large quantities and with alcohol. However, the sale and use of energy drinks remain unregulated in many developing countries such as Ghana.

Producers of energy drinks usually target young adults who are easily lured to consume energy drinks after watching numerous appealing marketing advertisements on television and in newspapers and magazines. However, concerns have been raised regarding the ingredients in energy drinks and their potential negative effects on people's health [16]. Although it has been reported that athletes are increasingly using energy drinks because of the ergogenic effects of caffeine and the other ingredients found in these beverages [16], research into energy drink consumption practices among young adults who actively participate in sports in most developing countries is almost non-existent. In addition, literature about the consumption of energy drinks by young people in Ghana is scarce despite the upsurge of different brands of energy drinks in shops dotted on university campuses in Ghana. Therefore the purpose of this study was to determine (1) the energy drink consumption practices among student-athletes, (2) the prevalence and frequency of intake of energy drinks and (3) reasons why athletes consume energy drinks. In the current study, an energy drink is defined as a kind of soft drink, which is usually carbonated and contains caffeine, sugar or other stimulants believed to reduce or prevent fatigue, provide energy, promote alertness and enhance one's physical performance. Findings of this study will be useful to sports managers and coaches who need to be aware of the consumption practices of their athletes to be able to impart knowledge of the health implications of excessive intakes of energy drinks and also correct misconceptions regarding the purported benefits of energy drinks.

## Methods

### Subjects

In this cross-sectional study, the study participants were university student-athletes sampled from seven public universities in Ghana. The respondents completed a questionnaire administered during an inter-university sports competition. Out of the 250 questionnaires which were distributed to the athletes, 180 athletes completed the questionnaire, resulting in a response rate of 72%.

### Study instrument and data collection

The questionnaire was in two parts, the first part assessed the socio-demographic characteristics of the respondents and the second part assessed energy drink consumption practices of the athletes and reasons why students consumed them. The questionnaire which was administered assessed athletes in the following areas: background information (i.e. age, gender, university affiliation and sports discipline), information on energy drink consumption practices, brands of energy drinks usually consumed and reasons why athletes consumed energy drinks.

The researchers explained to the study participants that the investigation was mainly aimed at assessing how and why energy drinks were consumed, a situation that had not been studied comprehensively among student- athletes in Ghana and that the findings would serve as a basis to plan and implement nutritional and health educational programmes for student-athletes. To ensure compliance and allay any kind of anxiety, the introduction informed students that all responses will be treated with great confidentiality and the data was solely for research purposes.

### Statistical analysis

Data collected were entered and analysed using the Statistical Package for the Social Sciences (SPSS) programme, version 16.0. Descriptive statistics were run to summarize the data collected and the results were displayed in frequencies and percentages. Differences between males and females in respect of frequency of intake were also assessed by conducting a Chi-Square test. Again responses given by the five different sports discipline groups (short distance, middle distance, long distance, team events and both field and track events) regarding frequency of intake were compared between the groups.

## Results

### Background information of study participants

The background information of the study participants is presented in Table 1. The study population comprised 82.2% males. A high proportion (46.7%) of the study participants were within the age category of 21 to 23 years. The majority (63.9%) of the study subjects participated in team events, rather than the other events. Out of the 180 respondents, only 19(10.6%) indicated that they had completed a nutrition-related course in the university. A majority (38.3%) trained for a period of between 1 and 2 hours in a day. The rest trained for longer periods per day.

**Table 1:** Background Characteristics of Study Participants

Variable	Groups	n (%)
Gender	Male	148(82.2)
	Female	32(17.8)

Age Group (years)	18-20	23(12.8)
	21-23	84(46.7)
	24-26	48(26.7)
	27-29	16(8.9)
	> 29	9(5.0)

University Affiliation	UG	32(17.8)
	UCC	42(23.3)
	UDS	22(12.2)
	UEW	26(14.4)
	KNUST	25(13.9)
	UMaT	10(5.6)
	IPS	23(12.8)

*Type of Sports Discipline	Short distance	30(16.7)
	Middle distance	17(9.4)
	Long distance	9(5.0)
	Team events	115(63.9)
	Both Track and Field events	9(5.0)

Completed a Nutrition Course in the University	Yes	19(10.6)
	No	161(89.4)

Training Hours per Day	1- 2 hours/day	69(38.3)
	3-4 hours/day	47(26.1)
	5-6 hours/day	64(35.6)

*Type of Sports Discipline: **Short Distance **- Athletics events ranging from 100 m to 400 m; **Middle Distance **- Athletics events ranging from 800 m to 1500 m; **Long Distance **- Athletics events ranging from 3000 m to 10000 m; **Team Events **- Comprises games like soccer, volleyball, basketball, hockey, badminton, tennis, table tennis and handball; **Both Track and Field Events **- Athletics events ranging from 100 m to 10000 m and field events which comprises athletics events like javelin, shot putt, discus, high jump, long jump, triple jump and pole vault

### Responses regarding energy drink consumption patterns

The prevalence regarding energy drinks consumption among the surveyed athletes was 62.2%. This is the percentage of athletes who reported consuming an energy drink in the week prior to the study and usually consumed at least one can of energy drink per week, as shown in Table 2. A high proportion (53.6%) of the respondents indicated that they usually drank Lucozade. Other brands of energy drinks consumed included Blue Jeans (16.1%), Red Bull (9.8%), Burn (8.9%), Rox (8.0%) and Gluconade (3.6%). The majority (79.5%) of the respondents reported that they usually drank between 1 and 2 cans of energy drink in a week, whereas 20.5% indicated that they drank between 3 and 4 cans of energy drinks per week.

**Table 2:** Energy Drinks Consumption Practices of Student-athletes

Variable	n (%)
**Consumption of energy drinks**	
Yes	112(62.2)
No	68(37.8)
**Type usually drank**	
Gluconade	4(3.6)
Burn	10(8.9)
Blue Jeans	18(16.1)
Rox	9(8.0)
Red Bull	11(9.8)
Lucozade	60(53.6)
**Approximate number of cans consumed per week**	
1-2 cans/week	89(79.5)
3-4 cans/week	23(20.5)

Reasons given as to why student-athletes consume energy drinks are shown in Table 3. A majority of the respondents (58.9%) indicated that they drank energy drinks because they helped one replenish lost energy. Other reasons given include the belief that energy drinks supply energy, replace lost body fluids (25.9%) and improve one's performance (9.8%). A few respondents, 6(5.4%), indicated that they drank energy drinks because they believed it helped in the reduction of fatigue.

**Table 3:** Reasons Why Student-athletes Drink Energy Drinks

Reason(s) for use	No. (%) of users
Provides energy and replaces body fluids losses	29(25.9)
Reduces fatigue	6(5.4)
Improves performance	11(9.8)
Replenishes lost energy	66(58.9)
Total	112(100)

### Comparison between male and female respondents regarding intake of energy drinks

Analysis run to assess the difference between males and females with respect to the frequency of energy drinks consumed per week using the Chi-square test at an alpha (α) value of 0.05 yielded the following test results of continuity correction value = 2.56; degrees of freedom (df) = 1; with an associated significance value (Asymp. Sig.) = 0.110. The results indicate that the difference between the proportions of males and females with respect to the consumption of energy drinks (number of cans consumed per week) is not statistically significant.

### Comparison between disciplines regarding intake of energy drinks

**Figure 1:**
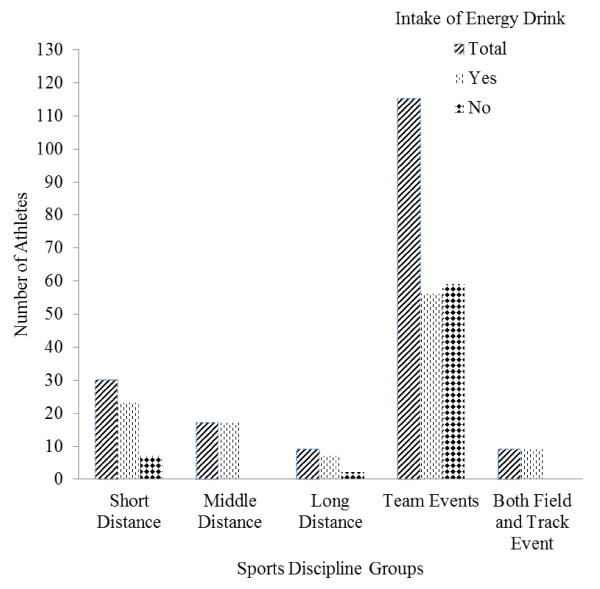
**Comparison between Sports Discipline Groups regarding Energy Drinks Intake in the Week before the Study**.

A comparison between the different discipline categories with regard to whether they drank energy drinks in the past week or not is shown in Figure 1. The results indicated that apart from team events athletes, a higher proportion of respondents belonging to the various discipline groups drank at least a can of energy drink in the week prior to the study. All middle distance athletes and athletes who participated in both field and track events reported that they took in some energy drink in the past week before the study.

**Figure 2:**
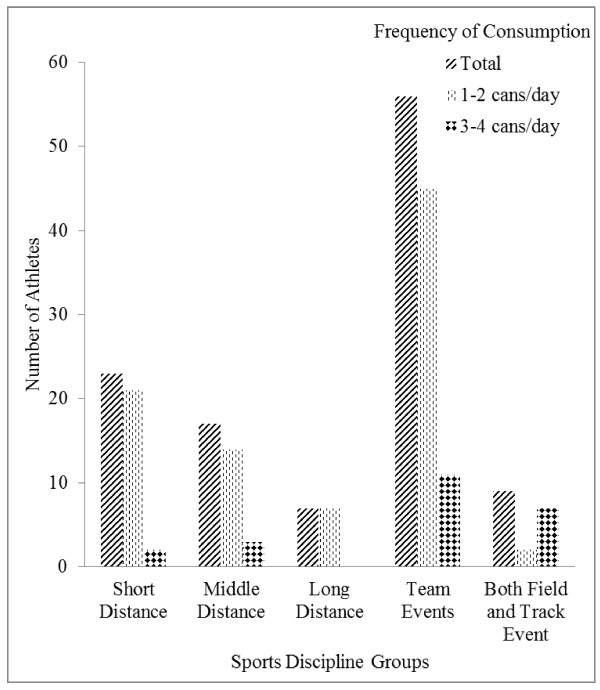
**Comparison between Sports Groups regarding Frequency of Energy Drinks Intake per Week**.

Regarding the frequency of consumption, a higher proportion of respondents who participated in both field and track events, reported that they usually drank between 3 and 4 cans of energy drink per week, as shown in Figure 2. A Chi-Square test was run to assess the difference between the sports discipline categories with respect to the frequency of consumption of energy drinks per week. The test at an alpha (α) value of 0.05 yielded the following test results; a Pearson Chi-Square value = 8.106; df = 4 with an associated significance value (Asymp. Sig.) of 0.001. This is an indication that the difference between the proportions of athletes belonging to the different sports discipline categories in relation to the number of cans of energy drinks consumed per week is statistically significant.

Regarding the reasons why energy drinks are consumed, results comparing between the different sports discipline groups is presented in Table 4. The results revealed that for 4 groups (short distance, middle distance, long distance and team events) athletes usually consume energy drinks because they believed energy drinks helps in replenishing lost energy. However, for respondents who participated in both fields and track events, a higher proportion reported that they usually drink energy drinks because it helps improve their performance.

**Table 4:** Comparison between Sports Discipline Groups regarding Reasons Why Energy Drinks are Consumed

Athletic disciplines	Reasons why energy drinks are consumed			
	
	Provides energy and fluids *(n = 29)*	Reduces fatigue *(n = 6)*	Improves performance *(n = 11)*	Replenishes lost energy *(n = 66)*
Short distance	3(13.0)	1(4.3)	0(0.0)	19(82.7)
Middle distance	2(11.8)	0(0.0)	2(11.8)	13(76.4)
Long distance	0(0.0)	0(0.0)	0(0.0)	7(100.0)
Team events	22(39.3)	3(5.4)	5(8.9)	26(46.4)
Field and track events	2(22.2)	2(22.2)	4(44.4)	1(11.2)

## Discussion

Generally, the current study indicated that energy drink consumption is a popular practice among athletes in the universities in Ghana. Most of the participants (62.2%) reported consuming at least one can of energy drink in a week similar to the finding of Ballistreri and Corradi-Webster [13] that 64.9% of the study participants consumed energy drinks. However, the percentage in the present study is slightly lower than in previous studies where higher proportions, 73% [17] and 86.7% [18] were reported. A lower prevalence value of 51% among surveyed college students in general was reported in a study by Malinauskas et al. [1]. Malinauskas et al. [1] further indicated that student-athletes in particular consumed energy drinks at a higher rate, seeing that many marketing advertisements linked energy drinks to sports.

A common reason given by most (64.1%) respondents regarding why they drank energy drinks was to help replenish lost energy after training sessions and competitions. Such a response is not surprising, for as asserted by Bonci (2002) [19], most people believe that drinking energy drinks is a fast means of obtaining 'extra energy' to undertake the activities of a day and speed up recovery from exercise. The findings of the present study corroborate those of Malinauskas et al., [1], in which 65% of college students indicated that they drank energy drinks because they needed energy. Similarly, Oteri et al. [20] reported that energy drink usage has become widespread among college students, particularly student-athletes who have to meet both cognitive and physical performance demands. Duchan et al. [16] also pointed out that young athletes are increasingly using energy drinks because of the ergogenic effects of caffeine and the other ingredients in these beverages which manufacturers claim as energy boosters.

Approximately 25.9% of the respondents also indicated that they consumed energy drinks because they provided energy and fluids to the body. However, it has been pointed out that there are serious consequences of substituting energy drinks for water when engaging in strenuous physical activities. This is because the caffeine in most energy drinks can have a dehydrating effect on the body. Caffeine acts as a diuretic agent and as such causes the kidneys to remove extra fluid from the body [6]. Consequently, if a person consumes energy drinks while sweating, it will result in severe dehydration. Therefore, energy drinks used during exercise or other strenuous activities compound the problem of dehydration, and do nothing to provide the body with any fluids. High consumers are at an even higher risk of sweating more and burning out all the extra energy supposed to have been obtained from the energy drinks. One can infer from the responses of the study participants that they are confused between the role of sports drinks and that of energy drinks. Unlike energy drinks, the purpose of sports drinks is to replenish lost body fluids, essential minerals and nutrients during and after an exercise.

Only 9.8% of the athletes indicated that they consumed energy drinks because they improved their performance. Literature available presents contradictory evidence regarding the capacity of energy drinks to enhance performance in sports. As indicated by Paddock [3], many of the marketing campaigns explicitly state that energy drinks help to improve the functioning and performance of an individual, suggesting that their consumption will boost athletic performance. A study indicated that the main ingredients in energy drinks support manufacturers' claims of an increased performance, endurance, concentration and an enhanced mood during physical activities [21]. Similarly, Janzen [22] pointed out that caffeine, a stimulant, increases alertness and enhances performance of certain tasks when consumed in small doses. In addition, Desbrow and Leveritt [23] reported that most elite athletes consume energy drinks in order to improve their physical performance and concentration during an activity. Other experimental studies revealed that, energy drinks increased long-term exercise endurance and improved speed and work output compared to a placebo drink [24,25]. Alford et al. [24] showed that consumption of energy drinks delayed the time of exhaustion in a study where the effect of energy drink on endurance performance was compared with carbonated water. Similarly, Mucignat-Carette [26] reported that a faster reaction time was observed in study participants who consumed energy drinks compared to participants who drank a placebo drink under similar controlled experimental conditions. Geiss et al. [27] also observed an improvement in the performance of athletes who consumed 500 ml of energy drink compared to the control group.

A comparison between energy drink consumption practices of males and females, shows that a higher proportion (81.2%) of the respondents who indicated that they drank energy drinks were males compared with 18.8% females. However, it is important to note the wide gender disparity (148 males to 32 females) in the study sample. In addition, whereas none of the females drank more than 2 cans of energy drink a week, all the respondents who drank more than 2 cans a week were males and represented 25.3% of the male population of energy drink consumers. The findings of this present study corroborate those of similar studies in which it was found out that male athletes consumed more servings of energy drinks than females [11,28]. Similarly, in another study, male-athletes indicated deliberately using energy drinks as stimulants and ergogenic aids [29]. A reason that can be given for the higher intake of energy drinks among males compared with females is perhaps, as asserted by Miller [11], advertisements of energy drinks which usually target primarily young adult males. Miller [11] further reported on the basis of a survey of undergraduate students that males (who reported that they employed measures to enable them appear more masculine in appearance) were more likely to increase their frequency of energy drink consumption. Furthermore, McClelland et al. [30] asserted that there are personality factors that determine the competitiveness of an individual, and that the need to achieve and the tendency to achieve success are more predominant in males than females. Most men are competitive, accept challenges, tend to be stimulated by situations involving task or role accomplishment and assume risks compared with females. These reasons could explain the high tendency for male athletes to consume energy drinks more often and in higher quantities than female athletes.

The health implications of an excessive intake of energy drinks, particularly brands that contain high quantities of caffeine, are numerous. High intakes of caffeinated drinks can result in irregular heartbeats, nausea, restlessness, headache, and dehydration [31]. One of the negative effects of energy drinks which contain high percentages of carbohydrates is that they often slow down the rate at which nutrients are absorbed into the bloodstream. Consequently, one's energy level is not likely to be boosted very much. In addition, a high quantity of carbohydrates slows down the rate of fluid absorption or rehydration during an exercise. Ingesting high levels of sugar can also lead to a high sugar crash. This occurs when sugar enters the blood stream and provides a "blast" of energy enabling the athlete to feel good and perform well. Once that energy is burned up, usually in about 30 to 45 minutes, there is a sugar crash. The athlete's reflexes slow down, causing dizziness and resulting in a decrease in muscle power and a subsequent drop in performance [32]. Other health implications include reported cases of seizures and cardiac arrest (following energy drink consumption) and dental enamel erosion resulting from the acidity of energy drinks [16].

In each of the sporting disciplines, except team events, a higher proportion of the study participants took energy drinks. In addition, a higher proportion of long distance and middle distance runners, compared with short distance runners, indicated that they consumed energy drinks. The findings also suggest that a higher proportion of middle distance runners, long distance runners and athletes who actively participate in both track and field events are more likely to consume energy drinks than athletes who participate in only team events and short distance disciplines. Most athletes in the team events group (with the exception of athletes who run as a team in track events) did not drink energy drinks, perhaps because these team events, by their nature, require explosive reactions, coupled with maximum strength, power and techniques rather than sustained energy levels. Therefore consuming energy drinks can offer little or no assistance to athletes who participate in these team events with respect to athletic performance. Also, the duration and intensity of team events can influence the decisions of athletes not to consume energy drinks frequently and in great quantities. It is known that middle and long distance events require sustained energy levels throughout the events (running at times between moderate to high intensity levels that could last for 40 minutes, an hour or beyond, with minimal or no rest intervals) compared with team events in which sustained energy periods for athletes are of short durations (with intermittent rest intervals), which may necessarily not require the consumption of energy drinks.

## Conclusions and suggestions for further study

Consumption of energy drinks is a popular practice among university student-athletes in Ghana, as 62.2% of the study participants reported that they drank at least a can of energy drink in the week prior to the study. Approximately 20.5% of the consumers who were all males drank between 3 and 4 cans per week. Most of the student-athletes who drank energy drinks indicated that the main reason why they drank energy drinks was to help replenish lost energy. Some athletes had wrong perceptions regarding the benefits of energy drinks which include its ability to help replace lost body fluids, improve one's performance and reduce fatigue when participating in any physical activity. Obviously, these wrong perceptions are as a result of the ignorance of students about the proven positive benefits and negative effects of energy drinks. The results suggest the need to create awareness through health education to prevent the consumption of energy drinks in excessive quantities and correct some wrong perceptions that athletes have regarding the benefits of energy drinks. In sum, the fact that the practice of consuming energy drinks is highly prevalent among student-athletes, particularly because they have ready access to an ever-increasing range of energy drinks, warrants the creation of continued public health awareness about the appropriate use of caffeinated beverages, their potential benefits and their side effects.

Suggestions for further studies include assessing whether students have any knowledge of the active ingredients in energy drinks and whether they have the right information about the potential positive and negative effects of the consumption of energy drinks.

## Competing interests

The authors declare that they have no competing interests.

## Authors' contributions

CB conceived the idea of the study, participated in the design of the study, analysis of data, drafted the first version of the manuscript and participated in finalizing the manuscript. EH participated in the design of the study, and had the major responsibility of recruiting subjects and coordinating the data collection and analysis of the data. He participated in developing the manuscript, discussing the findings and in finalizing the manuscript. Both authors gave suggestions, read the manuscript carefully, fully agreed on its content and approved its final version.
